# Immersive Surgical Anatomy of the Craniocervical Junction

**DOI:** 10.7759/cureus.10364

**Published:** 2020-09-10

**Authors:** Vera Vigo, Ankit Hirpara, Mohamed Yassin, Minghao Wang, Dean Chou, Pasquale De Bonis, Adib Abla, Roberto Rodriguez Rubio

**Affiliations:** 1 Neurological Surgery, University of California San Francisco, San Francisco, USA; 2 Neurological Surgery, First Affiliated Hospital of China Medical University, Shenyang, CHN; 3 Neurological Surgery, University of Caifornia San Francisco, San Francisco, USA; 4 Neurological Surgery, Ferrara University Hospital, Ferrara, ITA

**Keywords:** craniocervical junction, atlas, axis, occipital bone, biomechanics, cruciform ligament, volumetric model, neuroanatomy, surgical lines

## Abstract

With the advent and increased usage of posterior, lateral, and anterior surgical approaches to the craniocervical junction (CCJ), it is essential to have a sound understanding of the osseous, ligamentous, and neurovascular layers of this region as well as their three-dimensional (3D) orientations and functional kinematics. Advances in 3D technology can be leveraged to develop a more nuanced and comprehensive understanding of the CCJ, classically depicted via dissections and sketches. As such, this study aims to illustrate - with the use of 3D technologies - the major anatomical landmarks of the CCJ in an innovative and informative way. Photogrammetry, structured light scanning, and 3D reconstruction of medical images were used to generate these high-resolution volumetric models. A clear knowledge of the critical anatomical structures and morphometrics of the CCJ is crucial for the diagnosis, classification, and treatment of pathologies in this transitional region.

## Introduction

The craniocervical junction (CCJ) is a complex transitional region between the base of the skull and the upper cervical spine [1]. It is formed by the occipital bone and the first two cervical vertebrae, C1 or atlas and C2 or axis, both of which contain vital neural and vascular structures (i.e. brainstem, spinal cord, cranial nerves, and the vertebral artery). This articulation is the most mobile of the cervical spine and allows for 40% of all cervical flexion-extension and 60% of all head rotation [2]. The CCJ’s unique conformation of bones, ligaments, and muscles guarantees maximum stability of the head and generates a high degree of mobility. A layered understanding of the anatomy of the CCJ structures, as well as regional kinematics, is crucial to assess and treat the different diseases of this region, which can range from congenital malformations, traumatisms, inflammatory diseases, and tumors. Since the CCJ surrounds many critical structures, early identification of any possible disorder is essential to avoid severe neurological injuries. Understanding the correct alignments of the CCJ components and the relevant osteometric lines and angles is critical to select the best treatment, assess the best operative plan, and detect and classify the anomalies and instabilities of this region.

In this anatomical study, we aim to describe the anatomy, kinematics, and osteometry of the CCJ. Five embalmed cadaveric heads, two dry skulls, and a dry upper cervical spine were used to obtain images and volumetric models (VMs), which provide a unique, 360-degree visualization of the CCJ.

## Technical report

Materials and methods

Relevant anatomy of the CCJ - including osseous, muscular, ligamentous, and neurovascular anatomy - was depicted using five embalmed, latex-injected cadaveric heads and two dry skulls. The dissections were performed using a surgical microscope (OPMI Pentero 900; Zeiss, Oberkochen, Germany), and images were taken using a high-definition camera (D810; Nikon, Tokyo, Japan). VMs of the specimens were generated using photogrammetry and structured light scanning [3]. To reconstruct anatomical concepts, three base meshes were acquired from two 3D media repositories: BodyParts 3D (The Database Center for Life Science, University of Tokyo, Tokyo, Japan), and Sketchfab (Sketchfab Inc., New York, NY, USA). All the meshes underwent further geometrical postprocessing with Blender 2.8 (Blender Foundation, Amsterdam, the Netherlands) and ZBrush 2020.1.3 (Pixologic Inc., Los Angeles, CA, USA). Finally, photorealistic based materials and textures were edited using Substance Painter 2020.2.1 (Adobe, San Jose, CA, USA). No IRB/ethics committee approval was needed for our study.

Virtual platform

We used a web-based, 3D model viewer app (Sketchfab; Sketchfab Inc.) to upload our VMs, allowing for a truly immersive and interactive experience with the anatomy of the CCJ. The VMs with relevant annotations were then rendered in real-time, and the lighting and positioning of the models were manipulated to highlight anatomical regions of interest. The following instructions can be used to manipulate all models: to move, left click and drag; to zoom in and out, use the mouse scroll. For smartphones and virtual reality (VR)-ready computers, click "view in VR" (glasses icon); to view annotations, click on the numbers, to move around the object, tap or press trigger on the floor using the blinking yellow circle as a pointer. For mobile augmented reality (AR), click on the AR icon (cube) in the top right corner and aim at a horizontal flat surface; once the surface is detected, tap on it to place the model. To view the video in virtual reality mode, Google Cardboard, and YouTube mobile app are necessary. First, open the video on the YouTube mobile app and tap the Cardboard icon. Next, place the mobile device inside the Google Cardboard. Finally, look around to view the video in 360-degrees. Quality of the textures and navigation style can be modified by clicking the Settings icon.

Bones

**Figure 1:**
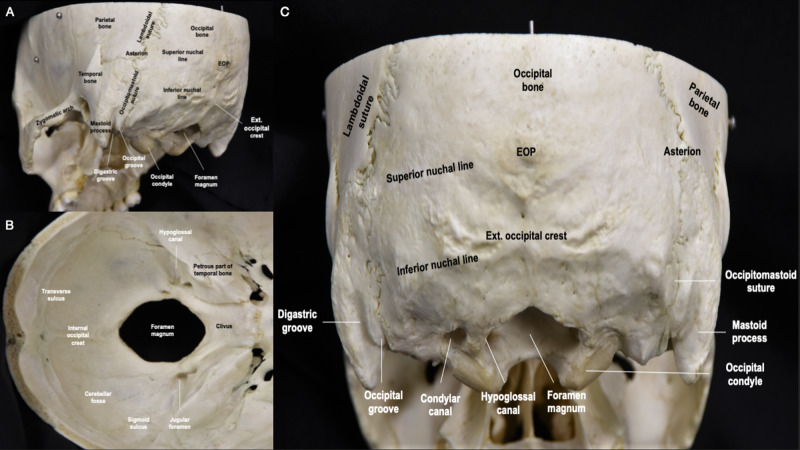
Overview of the occipital bone anatomy. (A) Posterolateral perspective of the skull illustrating the conical protuberance of the temporal bone, known as the mastoid process, and zygomatic arch; (B) Inferior perspective of the skull­ showing various grooves, canals, and corridors; (C) Posterior perspective of the skull revealing the reniform occipital condyles and their oblique orientation Occi. bone: occipital bone; EOP: external occipital protuberance; occi. groove: occipital groove; occi. condyle: occipital condyle; sup. nuchal line: superior nuchal line; mastoid proc.: mastoid process; ext. occi. crest: external occipital crest; int. occi. protuberance: internal occipital protuberance; int. occ. crest: internal occipital crest; trans. sulcus: transverse occipital sulcus.

The occipital bone is a trapezoid-shaped and curved bone that forms part of the skull base. ­It has an inner concave surface and an outer convex surface. The external surface is divided into four regions - squamous, two laterals (left and right), and basilar. Inferiorly, the occipital bone has a circular opening known as the foramen magnum, through which the spinal cord passes (Figure 1A-1C). The posterior edge of the foramen magnum (opisthion), which attaches to the atlantooccipital membrane, is located at the anteroinferior part of the squamous region. The inferior, superior, medial, and in some cases, the highest nuchal line, are localized posteriorly and superiorly to the opisthion. These lines are important muscular and ligamentous landmarks and play a crucial role in the mobility and stability of the CCJ (see below). The inion is a palpable protuberance of the squamous surface, which represents the projection of the internal occipital protuberance (Figure 1C). Importantly, the inion serves as a landmark for locating the junction of the transverse sinuses and the torcula Herophili, which is the confluence of the superior sagittal sinus, straight sinus, and occipital sinus.

The two lateral surfaces of the occiput are located on each side of the foramen magnum and are composed of the occipital condyles, jugular processes, and jugular notches. The condyles are two convex structures that protrude inferiorly, anteriorly, and medially, allowing for articulation with the superior facets of the atlas and thereby also allowing for cranial flexion, extension, and lateral bending (Figure 1A, 1C) [2]. This articulation is known as the atlantooccipital joint. The hypoglossal canal can be identified at the junction of the posterior and middle third of each condyle (Figure 1B, 1C). This short canal starts on the inner surface of the occiput, immediately above the foramen magnum, and has an oblique course, going from posterior and medial to anterior and lateral. The jugular process is a quadrilateral plate of the occiput, which extends laterally from the posterior half of each condyle and articulates with the temporal bone. The jugular notch is a groove along the lateral margin of each process that forms the posterior part of the jugular foramen (Interactive Model 1).

**Video 1:** Volumetric model of occipital bone, with annotations of the main structures.

The basilar portion of the occipital bone extends anteriorly and superiorly from the anterior part of the foramen magnum (basion), articulating with the basilar surface of the sphenoid bone to form the clivus. In addition to forming the anterior portion of the foramen magnum, the clivus is also a horizontal support structure for the pons and serves as a critical landmark for proper atlantooccipital alignment.

**Figure 2:**
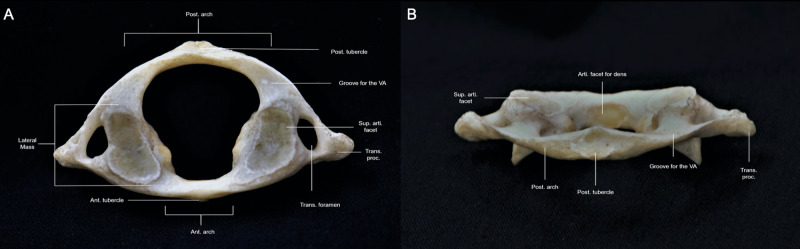
Overview of the atlas. (A) Superior perspective of the atlas revealing the concavity of the superior articular facets and the unique shape of the transverse foramen (B) Posterior perspective of the atlas revealing the concavity of the posterior tubercle and angle of the posterior arch. Post. arch: posterior arch; Post. tubercle: posterior tubercle; VA: vertebral artery; super. arti. facet: superior articular facet; trans. proc.: transverse process; trans. foramen: transverse foramen; ant. arch: anterior arch; ant. tubercle: anterior tubercle; arti. facet for dens: articular facet for dens.

The first cervical segment, also known as the atlas, is a ring-like vertebra that supports the occiput. The atlas has two arches that circumscribe the vertebral canal, both of which are separated by lateral masses with transverse processes projecting laterally from each side (Figure 2A-2B; Interactive Model 2). The significantly smaller anterior arch has a protrusion known as the anterior tubercle, which is an attachment point for the anterior longitudinal ligament and the superior oblique fibers of the longus colli muscle. The anterior arch also has a posterior facet that articulates with the odontoid process, which is a superior projection of the axial vertebral body (Figure 2B; Interactive Model 2) [4]. This articulation is further supported by the transverse ligament of the atlas, which bounds and constrains the dens posteriorly and prevents it from folding into the brainstem during flexion [1]. In addition to its articulation with the dens, the atlas also inferiorly articulates with the superior facet of the axis through its caudal and medial facets. The atlantoaxial articulation is responsible for most of the cervical spine’s rotation [1-2].

**Video 2:** Volumetric model of atlas, with annotations of the main structures

Most of the atlas circumference is formed by the posterior arch, which has a tubercle that gives attachment to the rectus minor muscle and the nuchal ligament. The inferior border of the posterior arch gives insertion to the ligamenta flava. On the lateral surface of the posterior arch, behind the superior articular process, lie two grooves that are surpassed by the vertebral artery (VA; third segment -V3) and the ipsilateral cervical dorsal ramus of C1, which travels anterior to the VA. The lateral mass of the atlas has a medial bony bump, known as the colliculus atlantis, that serves as the insertion point of the transverse atlantal ligament [5]. Posterior to the colliculus atlantis is the sulcus atlantis, which harbors a branch of the VA that supplies the ipsilateral lateral mass. Compared to the rest of the cervical vertebrae that have an anterior and posterior tubercle originating from the transverse process, the atlas only has one tubercle, which serves as a palpable landmark for the mastoid tip and mandibular ramus. The superior articular processes have a concave and medially rotated surface that articulates with the occipital condyle to form the atlantooccipital joint. 

Osseous variations of the atlas must also be taken into account. The two grooves for the V3 segment of the VA may be partially or entirely enclosed by calcification of the atlanto-occipital ligament, known as ponticulus posticus. The prevalence of this morphological feature is between 1.14% - 37%, and it transforms the groove of the VA into a canal known as the arcuate foramen. Another morphological variant is the occipitalization of the atlas, which is the incomplete segmentation between the atlas and occiput. This is prevalent in about 0.08% to 3.63% of the population. Lastly, about 2% of the population exhibits a defect in the posterior arch of the atlas due to the failure of local chondrogenesis, not due to osseous calcification, as seen in the case of ponticulus posticus [6]. This congenital defect presents itself as a posterior tubercle that joins with the axial, lateral masses.

**Figure 3:**
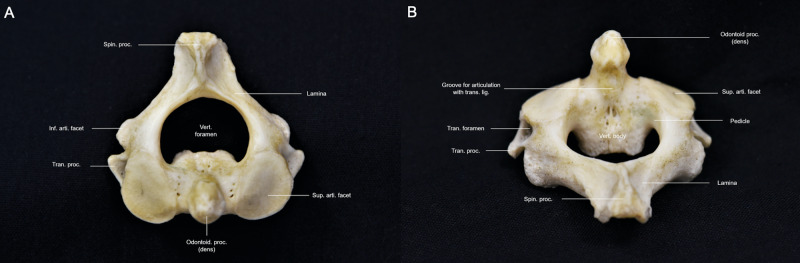
Overview of the axis. (A) Superior perspective of the axis revealing the bifid and large spinous process as well as the unique shape of the vertebral foramen; (B) Posterior perspective of the axis revealing the upward protuberance of the odontoid process and concavity of the superior articular facets Vert. foramen: vertebral foramen; odontoid proc.: odontoid process; sup. arti. facet: superior articular facet; inf. arti. facet: inferior articular facet; tran. proc.: transverse process; spin. proc.: spinous process; trans. lig.: transverse ligament; tran. foramen: transverse foramen

The second cervical vertebra, known as the axis, has a unique structure and is formed by the odontoid process, superior articular processes, and transverse processes (Figure 3A-3B; Interactive Model 3).

**Video 3:** Volumetric model of axis, with annotations of the main structures

The odontoid process, also known as dens or peg, protrudes superiorly and articulates posteriorly to the C1 anterior arch, forming a synovial joint. The tip of the dens serves as an insertion point for the apical ligament and, on each side, the alar ligaments. The fusion of the odontoid process with the body of C2 occurs via subdental synchondrosis. An unattached odontoid, known as an os odontoideum, can be identified on imaging when this fusion fails. The axis has much larger laminae compared to the rest of the cervical spine due to the transmission of forces applied superiorly [7]. Protruding from the laminae is a bifid spinous process, which serves as the attachment site for many muscles that connect to the occipital bone. Another anomaly of the axis is that its transverse processes, which protrude obliquely, superiorly, and laterally, lack distinct tubercles. The foramen transversarium is a gap in the transverse process through which the VA passes. The transverse foramen of the axis is positioned mediolaterally, which forces the VA to travel more laterally (Interactive Model 3). The pedicles of the axis form the superior articular processes, which articulate with the inferior articular processes of the atlas and form the atlantoaxial joints. The inferior articular processes of the axis face anteriorly, inferiorly, and laterally and form facet joints with the superior articular processes of C3.

Ligaments

**Figure 4:**
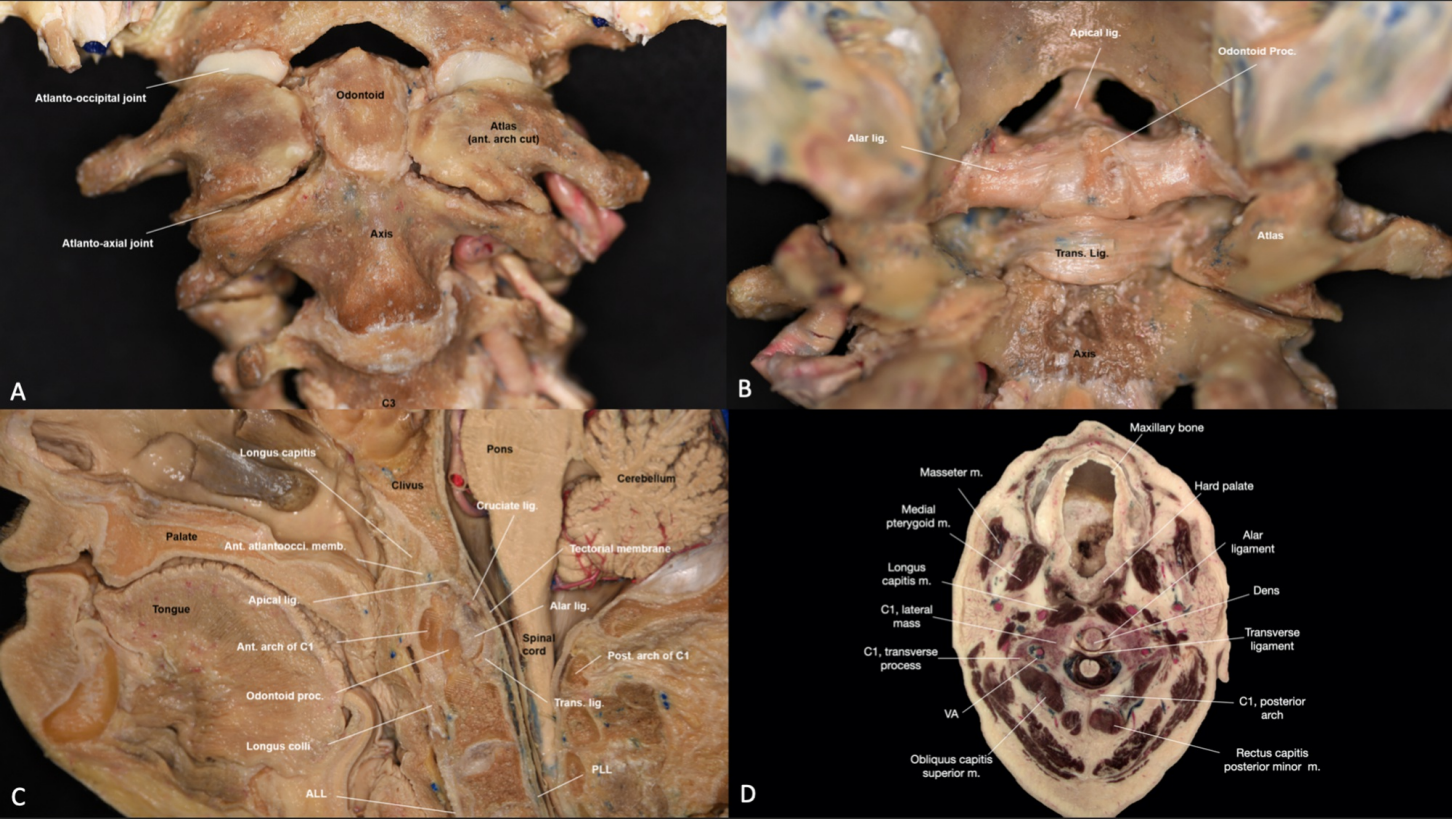
Overview of the main joints and ligaments of the craniocervical junction (CCJ) region. (A) Anterior view of the CCJ, after drilling the anterior arch of the atlas to show the dens (B) Posterior view of the CCJ joint and ligaments (C) Sagittal view of the CCJ (D) Axial view of the atlantoaxial joint. Ant. atlantoocci. memb.: anterior atlantooccipital membrane; apical lig.: apical ligament; ant. arch of C1: anterior arch of C1; odontoid proc.:  odontoid process; ALL: anterior longitudinal ligament; alar lig.: alar ligament; longus capitis m.: longus capitis muscle; mastoid m.: mastoid muscle; medial pterygoid m.: medial pterygoid muscle; obliqus capitis superior m.: obliqus capitis superior muscle; post arch of C1: posterior arch of C1; trans. lig.: transverse ligament; PLL: posterior longitudinal ligament; rectus capitis posterior minor m.: rectus capitis posterior minor muscle; VA: vertebral artery.

The anterior atlantooccipital membrane is the upper continuation of the anterior longitudinal ligament (Figure 4C). This membrane starts at the superior aspect of the anterior arch of the atlas and attaches to the basion. Together with the capsular ligaments of the atlantooccipital articulation, the anterior atlantooccipital membrane limits the extension of the occipital bone on C1 [2]. The posterior arch of the atlas is attached to opisthion through the posterior atlantooccipital membrane, which limits flexion of the occipital bone relative to the atlas. This structure is frequently adherent to the dura mater of the spine. The superior continuation of the posterior longitudinal ligament, also known as the tectorial membrane, begins at the posterior aspects of the vertebral body of C2 and inserts onto the basilar portion of the occipital bone and the basion, passing through the dens and all of the occipitoaxial ligaments. This ligament limits both flexion and extension of the atlantooccipital articulation [1]. The lateral atlantooccipital ligament is present bilaterally and is located posterior to the rectus capitis lateralis muscle. It is found alongside the anterior atlantooccipital membrane and begins at the anterolateral surface of the transverse process of the atlas and inserts onto the jugular process of the occipital bone.

The cruciform ligament, which gets its name from its cross-shaped structure, consists of the transverse ligament and the superior and inferior longitudinal fibers (Interactive Model 4). The transverse ligament holds the atlas in its correct orientation and allows it to pivot on C2 (Figure 4B, 4D). The ligament attaches to the lateral tubercles of the atlas and supports the atlantoaxial joint by holding the odontoid process of the axis against the posterior aspect of the atlas, specifically at the anterior arch. The longitudinal fibers attach the body of the axis to the clivus and foramen magnum. The superior longitudinal ligament goes from the transverse ligament to the basion and basilar portion of the occipital bone. It is located anterior to the tectorial membrane and posterior to the dens and in-between the apical ligament of the odontoid process. The inferior longitudinal fibers prevent the transverse ligament from migrating superiorly by attaching it to the body of C2.

**Video 4:** Volumetric model of the anterior ligaments of the CCJ

Though the left and right alar ligaments are similar in strength to the transverse ligament, they attach the axis to the left and right base of the skull (Figure 4B, 4D). Specifically, they connect the sides of the axis to the left and right tubercles of the occipital condyle.

The apical ligament of the odontoid process goes from the posterosuperior surface of the dens and attaches to the basion, merging with the superior longitudinal fibers of the cruciform ligament (Figure 4B, 4C). This ligament is located just posterior to the anterior atlantooccipital membrane and prevents anterior shear and vertical rotation of the occiput. The “apical cave” is a crucial area between the odontoid process and the basion, and it is covered by neurovascular structures (i.e. coiled venous plexus, recurrent meningeal branches of C1 and C2, and the apical odontoid arterial arcade) [7].

The transverse occipital ligament is an accessory ligament located posterior and superior to the alar ligaments and odontoid process. It attaches to the occipital condyles and extends across the foramen magnum. It is important to note, however, that this ligament is not always present and can be easily confused with the alar ligament due to similarities in morphology. When present, the transverse occipital ligament limits articulation at the atlantoaxial junction. The accessory atlantoaxial ligament is much more common and can be found inserting onto the dorsal aspect of the axis as well as on the posterior aspect of the transverse ligament [1]. Right behind the ligament is the tectorial membrane. All the ligaments of the craniocervical junction and their respective attachments and functions are listed in Table 1.

**Table 1:** Ligaments of the craniocervical junction and their respective attachments and functions

Location	Name	Insertions	Function
Posterior	Transverse Ligaments	Connnects the anterior surface of odontoid process with the posterior surface of the Atlas' anterior arch	Acts as the main stabalizer for the atlantoaxial joint and allows for rotation around this joint
	Alar Ligament	Connects the lateral sides of the odontoid process with the base of the skull	Acts as a stabalizer for the atlantoaxial joint and prevents excessive rotation around the joint
	Transverse Occipital Ligament	Located suprior to the bottom of the alar ligament and attaches to the inner walls of the Atlas' occipital condyles	Provides support to flexion and rotation of the head
	Barkow Ligament	Connects to the anterior aspects of the occipital condyles and wraps around the posterior edge of the odontoid process	Minimizes extension of the Atlantoaxial Joint
	Apical Ligament	Attaches the odontoid process with the basion of the foramen magnum	Provides minmal stabalization to the atlantoaxial joint
	Tectorial Membrane	Composed of three layers that run from the clivius to the body of the axis and connects laterally to the hypoglossal canals	Limits extension at atlanto-occpital joint and flexion at the atlanto-axial joint
	Posterior Atlanto-Occipital Membrane	Connects the inferior edge of the Atlas' posterior arch with the posterior edge of the foramen magnum	Minimal stability of the cranio-cervical junction
	Accessory Atlanto-Axial Ligament	Attaches to the dorsal surface of the Axis body and travels upwards and inserts posteriorly into the transverse ligament	Limits axial rotation of the head
Anterior	Anterior Atlanto-Occipital Membrane	Attaches to the superior edge of the Atlas' anterior arch and connects to the anterior edge of the foramen magnum	Limits extension of the atlanto-occipital joint
	Anterior Atlanto-Axial Membrane	Connects the inferior surface of the Atlas' anterior arch with the superior anterior surface of the Axis' body	Limits extension of the Atlanto-Axial joint
	Lateral Atlanto-Occipital Membrane	Connects the lateral surface of the Atlas' transverse process with the jugular of the occipital bone	Limits lateral flexion of the head

Muscles

**Figure 5:**
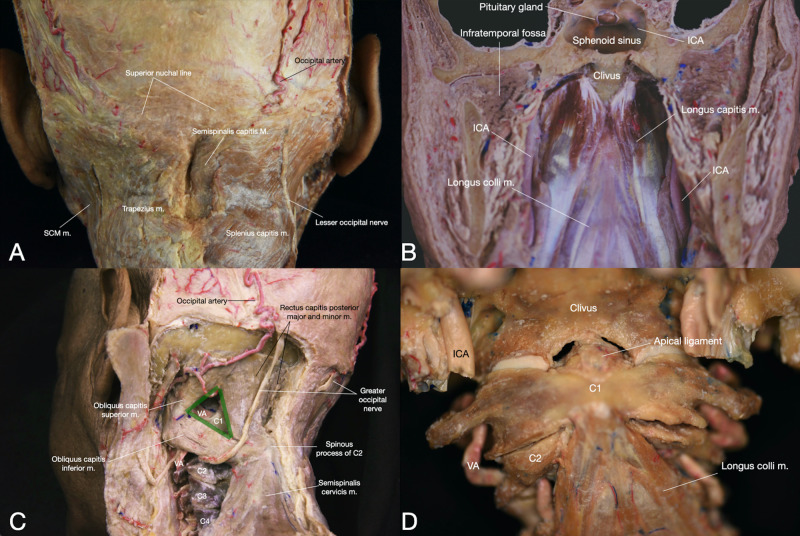
Overview of the main muscles of the craniocervical junction (CCJ). (A) Posterior view of the cervical muscles – superficial layer (B) Anterior view of the CCJ muscles (C) Posterolateral view of the cervical muscles - deep layer – the suboccipital triangle is highlight in green (D) Anterior view of the CCJ and the longus colli muscle. ICA: internal carotid artery; longus colli m.: longus colli muscle; obliqus capitis inferior m.: obliqus capitis inferior muscle; obliqus capitis superior m.: obliqus capitis superior muscle; rectus capitis posterior mayor and minor m.: rectus capitis posterior major and minor muscle; semispinalis cervicis m.: semispinalis cervicis muscle; semispinalis capitis m.: semispinalis capitis muscle; splenius capitis m.: splenius capitis muscle; SCM m.: sternocleidomastoid muscle; trapezius m.: trapezius muscle; VA: vertebral artery.

Muscles associated with the CVJ can be divided into the posterior (deep and superficial), lateral and anterolateral muscles groups (Table 2, Figure 5). Several suboccipital muscles control orientation and movements of the head (i.e., extension, flexion, abduction, adduction, and rotation) in relation to the craniocervical region. Anteriorly, these muscles are the rectus anterior and rectus lateralis, and posteriorly the rectus capitis posterior major and minor and the obliquus inferior and superior.

**Table 2:** Muscles of the craniocervical junction and their respective attachments and functions

Location		Muscle	Origin	Insertion	Function
Back Muscle	Suboccipital Deep Muscles	Rectus Capitis Posterior Major	Spinous process of C2	Lateral of inferior nuchal line on the occipital bone	Rotation and extension of head (through atlanto-occipital joint)
		Rectus Capitis Posterior Minor	Posterior arch of C1	Medial of inferior nuchal line on the occipital bone	Backward extension of the head
		Obliquus capitis superior	Transverse process of C1	lateral half of the inferior nuchal line on the occiptal bone	Act at the atlanto-occipital joint to extend or flex the head to the ipsilateral side
		Obliquus capitis inferior	Spinous process of C2	Transverse process of C1	Rotate the head and C1 vertebra through atlanto-axial joint
	Superficial Layer	Semispinalis Capitis	Articular Process of C5 - C8 and Transverse Process of T1 - T6	Between Superior and Inferior Nuchal Lines	Bilateral Extension and Horizontal Rotation
		Trapezius Muscle	Superior Nuchal Line of Occipital Bone	Clavicle and Scapula	Scapulary Movement and Spinal Stabalization
Lateral Muscles		Splenius Capitis	spinous process of C7	Mastoid process of the Temporal Bone	Rotation and Felxion of the Neck
		Longissimus Capitis	Trasnverse process of T1 - T4	Posterior of Mastoid Process of the Temporal Bone	Extension and Rotation of the Head
Anterolateral Muscle		Sternocleidomastoid	Manubrium, medial portion of clavicle	Mastoid process, superior nuchal line	Cervical rotation and flexion
		Longus Capitis	Anterior tubercle of the transverse process from C3 to C6	Inferior surface of basilar portion of occipital bone	Flexion of the neck at atlantoaxial joint
		Longus Colli	Transverse process of C3 through C6, anterior vertebral body of C5 to T3	Anterior arch of C1, anterior vertebral body of C2 through C4, and transverse process of C5 and C6	Flexing of cervical spine
		Scalene (anterior, middle, and posterior)	Transverse process of C2 through C7	First and second ribs	Flex and bend the neck laterally as well as elevate the first two ribw

The rectus capitis posterior major as well as the obliquus capitis superior and inferior constitute the suboccipital triangle (Figure 5C, Interactive Model 5). The posterior arch of C1 with the V3 segment of the VA and the C1 spinal nerve can be identified deep in this area. This triangle is a crucial landmark during posterior craniocervical surgery to early expose the VA. Other triangles to identify the VA have been described here as well. The subatlantic triangle is defined inferiorly and laterally by the levator scapulae and splenius cervicis muscles, medially and inferiorly by the longissimus capitis muscle, and superiorly by the inferior oblique capitis. The atlantoaxial portion of the VA is located deep in this triangle [8]. The inferior suboccipital triangle is limited by the inferior oblique muscle superiorly, the posterior intertransversarii muscle inferolaterally, and the C2 lamina inferomedially. Deep in this triangle, the lower part of the V3 segment laterally and C2 ganglion and rootlets medially can be recognized [9]. The condylar triangle is located superiorly to the suboccipital triangle and is formed by the rectus capitis lateralis anteriorly, superior oblique posteriorly, and the connection line between these two muscles along the occipital bone superiorly. This triangle encases the anterior third of the occipital condyle, the superior aspect of the genu of the V3 segment of the VA. Moreover, it provides a landmark for the terminal portion of the hypoglossal canal [10].

**Video 5:** Volumetric model of the bones, ligaments, muscles, and artery of the CCJ

Nerves

**Figure 6:**
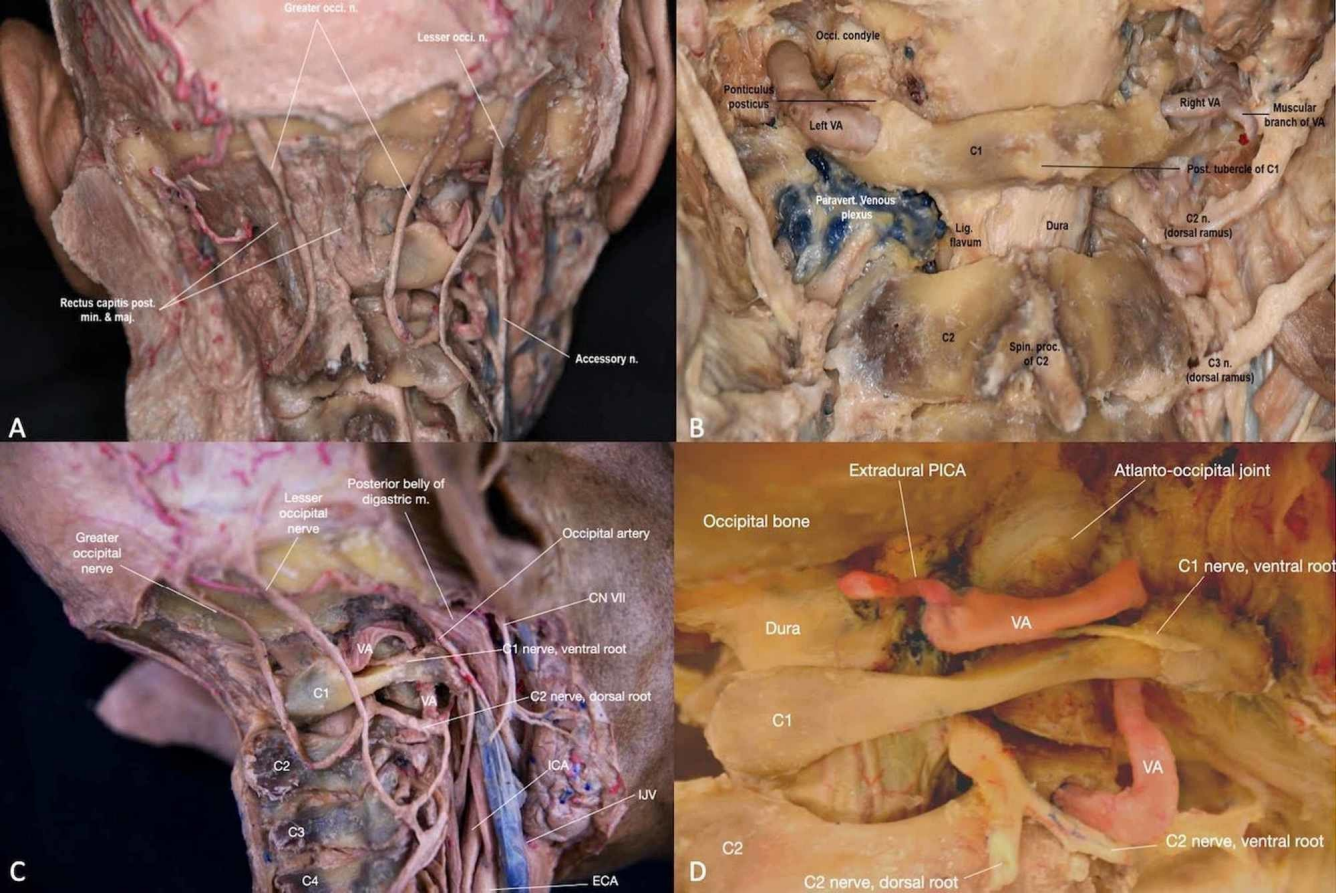
Overview of neurovascular anatomy of the craniocervical junction (CCJ) (A) Posterior view of the CCJ with attention to neural anatomy in the context of deep back muscles and cervical vertebrae (B) Close-up of the posterior view of the neurovascular structures of the CCJ (C) Lateral view of the CCJ with attention to major arteries, nerves, and veins (D) Close-up of the lateral view of the neurovascular structures of the CCJ. CN VII: facial nerve; C2 n. (dorsal root): C2 nerve (dorsal root); C3 n. (dorsal root): C3 nerve (dorsal root); Extradural PICA: extradural posterior inferior cerebellar artery; Greater occi. n.: greater occipital nerve; lesser occi. n.: lesser occipital nerve; lig. flavum: ligamentum flavum; rectus capitis post. min. & maj.: rectus capitis posterior minor & major; accessory n.: accessory nerve; posterior belly of digastric m.: posterior belly of digastric muscle; ICA: internal carotid artery; ECA: external carotid artery; paravert. venous plexus: paravertebral venous plexus; post. tubercle of C1: posterior tubercle of C1; occi. condyle: occipital condyle; spin. proc. of C2: spinous process of C2; IJV: internal jugular vein; VA: vertebral artery.

The first cervical nerve, known as the suboccipital nerve, exits superiorly to the posterior arch of the atlas and posteromedial to the lateral mass through a groove formed by the slight elevation of the superior articular surface of atlas. It shares this groove space with the VA, and the superior surface within the groove is lined by the posterior occipitoatlantal ligament (Figure 6D). The suboccipital nerve then travels to the suboccipital triangle in the neck and proceeds to send somatic motor fibers to the hypoglossal nerve, which innervates the extrinsic and intrinsic muscles of the tongue. It is important to remember that as nerves exit the spinal cord through intervertebral foramina, they divide into anterior and posterior nerve fibers.

The cervical plexus is a complex structure that begins with anterior primary rami from C1-C4. It is located in the posterior triangle of the neck, which includes the upper suboccipital triangle and lower subclavian triangle, and it innervates structures in the head, neck, and trunk of the body, such as the sternocleidomastoid and trapezius muscles. Specifically, the plexus can be found ventral and lateral to the levator scapulae and middle scalene muscles and deep relative to the internal jugular vein and sternocleidomastoid muscle. The cervical plexus can be conceptualized as two different plexuses, one being superficial and the other being deep. The superficial plexus is composed of lateral terminal branches that form loops, which contribute to the sensory branches of the cervical plexus and later become the great auricular nerve, transverse cervical nerve, and the lesser occipital and supraclavicular nerves. On the other hand, the deep plexus forms loops that contribute to the muscular branches of the cervical plexus and later become the ansa cervicalis, phrenic nerve, and different segmental branches [11].

The second cervical nerve exits between the posterior arch of the atlas and lamina of the axis, right above the pedicle of the axis and just below the obliquus inferior. Its posterior ramus happens to be the largest of all posterior cervical divisions. The most important nerve of the dorsal ramus of the C2 nerve is the greater occipital nerve, which is a purely afferent nerve derived specifically from the medial branch (Figure 6). It ascends past the suboccipital triangle and between the obliquus inferior and semispinalis capitis, after which it pierces the latter muscle and the trapezius, specifically near their attachments to the occipital bone (Figure 5C). It continues to ascend on the back of the head, passing the superior nuchal line and running alongside the occipital artery as well as the medial branch of the posterior aspect of the third cervical nerve. It then provides cutaneous branches to the skin of the nuchal region as well as muscular branches to the semispinalis capitis [12]. Additionally, the lesser occipital nerve is found lateral to the greater occipital nerve and deep relative to the splenius capitis muscle (Figure 6A, 6C). It ascends laterally to the semispinalis capitis, providing cuaneous branches to the region of the posterior scalp and occasionally the pinna of the ear (Interactive Model 6).

**Video 6:** Volumetric model of specimen with posterior cervical spine dissection showing the main neurovascular structures of the CCJ

Vascular

The vertebral arteries arise from the subclavian artery in pairs and travel upwards through the left and right transverse foramina of the cervical spine in order to supply the posterior region of the skull base, including the brainstem, cranial nerves, and cerebellum, as well as the occipital lobes and spinal column. The VA is posterior to the internal carotid artery, anterior to the hypoglossal nerve, and lateral to the uncinate process (Figure 6C). The VA can be divided into four segments: preforaminal (V1), foraminal (V2), atlantic (V3), and intradural (V4). The V1 segment begins at the posterosuperior aspect of the subclavian artery and continues only through the foramen of C6, and sometimes C5 or C7. The V2 runs through the foramen of C6 to C2, and it may be vulnerable to compression from osteophytes of the uncinate process. Retracting the rectus capitis posterior major and minor will reveal the C2 foramen, through which the V2 passes. After passing the foramen of C2, the VA makes a 45 degrees lateral turn (Figure 6D) and continues to pass through the foramen of C1, which marks the start of the V3 segment of the VA [13]. The V3 makes a posteromedial turn around the superior articular process of C1 and reaches the posterior arch of the atlas, where it runs along a groove known as the sulcus arteriosus. This region lies directly below the external occipital crest, an important marker to locate the position of V3. Recall that the suboccipital nerve also runs in this groove region. The V3 then passes inferiorly to the posterior atlantooccipital membrane and its ossified bridge, known as the ponticulus posticus (Figure 6B). Before passing through the membrane and into the foramen magnum, however, the VA runs horizontally about 1.67 cm from C1’s posterior tubercle [14]. The horizontal portion of the V3 is directly above the paravertebral venous plexus. The V4 segment continues anteriorly, superiorly, and medially from the posterior atlantooccipital membrane and rises along the anterolateral aspect of the spinal dura mater. The left and right V4 then pierce the dura and arachnoid mater while continuing to ascend within the subarachnoid space. It is important to note that as the VA traverses through the spinal column, it provides osteoarticular, meningeal, and spinal rami branches, which supply the vertebral body, posterior arch, and soft tissue structures within the vertebral canal. Both VA give a branch that joins its contralateral mate to form the single anterior spinal artery. This artery supplies the anterior spinal cord along all its length. Moreover, each VA (V4 segment) develops a posterior spinal artery that provides blood flow to the posterior columns of the spinal cord. Finally, the left and right V4 segments join at the base of the skull to form the basilar artery, which supplies the brain, brain stem, and cerebellum.

The occipital artery originates from the carotid artery and passes beside the transverse process of C1 and the mastoid process of the temporal bone to reach the occipital region of the head (Figure 6A, 6C). They are responsible for supplying blood to major muscle groups of the neck, such as the sternocleidomastoid muscle, and back of the scalp.

The right common carotid artery arises near the right sternoclavicular joint while the left common carotid artery arises directly from the aorta. They ascend up the neck, medial to the sternocleidomastoid muscle, and split into external and internal carotid arteries at the level of C3-C4. The external carotid artery travels posteriorly to the mandibular neck and anteriorly to the lobule of the ear and terminates within the parotid gland by dividing into the superficial temporal artery and maxillary artery. The internal carotid artery does not supply any of the structures in the neck but enters the cranial cavity through the carotid canal in the petrous part of the temporal bone (Figure 6C). The carotid canal is approximately 3 mm anteromedial to the transverse foramen of C1 [7].

The junction of the posterior auricular and posterior retromandibular veins forms the external jugular vein, which drains most of the scalp as well as the superior and lateral face region. The central face region, on the other hand, drains into the internal jugular vein, which runs lateral to the carotid artery.

## Discussion

Craniospinal morphometrics of clinical relevance

**Figure 7:**
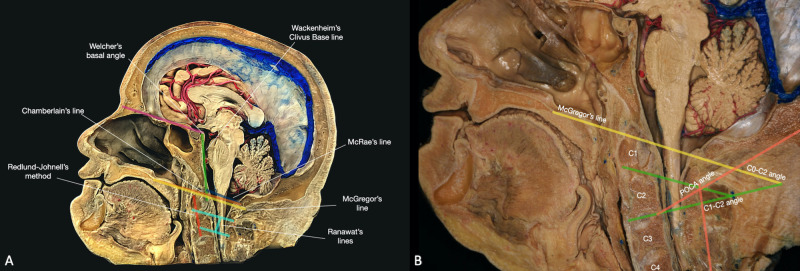
Overview of the CCJ morphometrics. (A) Anatomical lines and planes of the CCJ (B) Principles alignment angles of the CCJ. POCA: posterior occipitocervical angle.

The complex anatomy and the different lesions affecting the CCJ have increased the interest in this region in order to facilitate diagnosis and improve surgical treatment. For these reasons, multiple lines, planes, and angles have been characterized to assess the relationship between the structures of the CCJ. In this section, we will discuss the main morphometrical indexes that are used to detect and classify congenital or acquired anomalies and to estimate the best surgical route (Figure 7).

Anatomical Lines

· The Chamberlain Line goes from the posterior edge of the hard palate to the opisthion (Figure 7A). This line is used to identify basilar invagination when the odontoid process extends superiorly more than 6 mm (normal ± 3 mm). A modification of this line is the McGregor line, which goes from the posterior pole of the hard palate to the lowest portion of the squamous surface of the occiput (Figure 7A). This line is much easier to identify on standard imaging studies. The odontoid process should be located around 1.45 mm in males and 0.44 mm in females above the McGregor line. The basilar impression is defined for a distance greater than 4.5 mm [15].

· The McRae’s line determines the foramen magnum anteroposterior diameter, going from basion to opisthion (Figure 7A). The odontoid process should be located below this line [16].

· The Wackenheim’s Clivus Base Line is drawn along the clivus into the upper cervical canal (Figure 7A). The posterior aspect of the odontoid process should be tangent to this line. The intersection of the Wackenheim’s clivus baseline with a line drawn from the posterior aspect of the odontoid process to the inferodorsal surface of the axis body forms an angle (clivus-canal angle) that should range from 150° in flexion to 180° in extension. Angles less than 150° may cause ventral spinal cord compression [15].

· Ranawat’s line is a perpendicular line that connects the C2 sclerotic ring or base (Ranawat’s modified line) to a horizontal line drawn along the transverse axis of the atlas (Figure 7A). This line distance should be around 17 mm in males and 15 mm in females [16]. The Ranawat’s modified line should range between 31.1 ± 2.4 mm in males and 28.4 ± 2.1mm in females [17].

· The Redlund-Johnell method measures the length from the McGregor’s line to the midpoint of the axis base endplate (Figure 7A).

· The Welcher’s basal angle is determined by the junction between the nasion-tuberculum and the tuberculum-basion line (Figure 7A). This angle usually measures around 132°. Values above 140° denote an abnormally flattened skull base [15].

· The atlantooccipital joint axis angle is described by two lines drawn parallel to the atlantooccipital joints, which intersect at the center of the dens when the condyles are symmetrical. This angle is typically 125° (ranging from 124° to 127°), and it is obtuse in the event of occipital condyle hypoplasia [15].

· The Power’s ratio is calculated by dividing the distance basion-posterior ring of C1 by the distance opisthion-anterior ring of C1. This value should be equal to 1 or slightly less. Values higher than 1 denote anterior dislocation of the atlantooccipital joint, while lower than 0.7 suggest posterior dislocation (odontoid fracture or congenital narrowing of the occipital foramen) [2].

CCJ Alignment Angles

· The occipitoaxial angle (C0-C2 McGregor) is determined by the crossing of an oblique line drawn along the inferior endplate of the axis with the McGregor line (Figure 7B). This angle ranges between 14.8° ± 3.1° in males and 14.2° ± 4.3° in females [18].

· The posterior occipitocervical angle (POCA) is described as the intersection of a tangential line along the flat squamous portion of the occipital bone (from the foramen magnum to the external occipital protuberance) with a line formed by the posterior aspect of the C3 and C4 facets (Figure 7B). The values of this angle vary from 108.3°± 7.8° and 108.0° ± 8.4°, respectively in males and females [18].

· The C1-C2 or atlantoaxial angle is measured by the intersection of the lines parallel to the inferior aspects of C1 and C2 (Figure 7B). The average of this angle is in 26.4° ± 4.6° in males and 28.2° ± 4.0° in females [19].

· The C2-C7 Cobb’s angle, also known as the subaxial angle, is formed by the junction of the lines parallel to the inferior endplates of C2 and C7. This angle ranges in between 16.3° ± 7.3° in males and 12.7° ± 6.6° in females [19].

Surgical Lines

· The hard-palate line passes through the anterior and posterior edges of the palatine bone and meets the spine posteriorly (Interactive Model 7). The endoscopic endonasal approach (EEA) should be used if the CCJ lesion is above this line and the transoral approach (TA) if it is below the line.

**Video 7:** Volumetric model of the CCJ bones with drawing of the main surgical lines

· The nasopalatine line goes from the rhinion (which is the craniometric point for the most inferior portion of the nasal bones) to the posterior edge of the palatine bone (posterior nasal spine) (Interactive Model 7). Its continuation on the spine marks the inferior surgical limit of the EEA.

· To establish this inferior limit more accurately, two other lines have been described: the nasoaxial and nasopalatine lines. The nasoaxial line starts at the midpoint between the rhinion and the anterior nasal spine of the maxillary, while the rhinopalatine line starts from the two-thirds point between the rhinion and the anterior nasal spine. Both lines pass through the posterior edge of the palatine bone and reach the cervical spine posteriorly (Interactive Model 7). A wide-open mouth conventional radiograph is required to determine the superior and inferior surgical limit of the TA.

· The palatine inferior dental arch line (PIA) defines the superior limit of the TA, and it is drawn from the inferior dental arch to the posterior aspect of the hard palate. The surgical inferior dental arch line starts at the inferior dental arch and passes through the soft palate (Interactive Model 7). This line has been shown to be more reliable to determine the maximal extent of superior dissection rather than the conventional PIA [20].

· The atlantosuperior dental arch line represents the inferior limit of the TA and goes from the superior dental arch to the anterior base of the atlas (Interactive Model 7).

· Surgical landmarks of the CCJ surgery can be identified in the middle turbinate and the inter-Eustachian line, drawn between the two Eustachian tubes, that underline the atlantoaxial junction [16].

Kinematics and stability of the CCJ

The unique structure of the CCJ is designed to allow high degrees of motion and give stability and support to the head. The CCJ mobility is correlated to the visual and auditory exploration of space and can accomplish a range of motion of 33-47° in flexion/extension, 90° in rotation, and up to 12.2° in lateral bending (Interactive Model 8) [1]. The medial longitudinal fasciculus connects the vestibular nuclei with the extraocular muscles and XI cranial nerve nuclei, which control the eyes, head, and neck movements, respectively. To allow stereoscopic view while exploring the surrounding space, the CCJ has simultaneous independent movements around three axes: flexion-extension on a transversal axis, axial rotation around a vertical axis, and the combination of the two. Due to its high degree of mobility, the CCJ complex is composed of several muscular groups, a number of bone structures and ligaments which restrict movements and ensure stability on both joints, the atlantooccipital and atlantoaxial. The occiput-C1 junction allows for the majority of flexion-extension (25°). The sagittal displacement of the basion and the dens (which should not exceed 1mm) is avoided by the tectorial membrane, alar ligaments, and, in minor contribution, by the C1 facets. This joint also performs 5° of lateral bending and axial rotation. The alar ligament limits both movements, while the superior C1 facets aid in restraining the axial rotation. The atlantoaxial articulation allows for 15° of flexion-extension, limited by the tectorial membrane and dens-C1 arch contact, and 5° of lateral bending, limited by the alar ligament, which is also responsible for the forced rotation of C2. This joint performs most of the rotation of the head (40°), thanks to the rotation of the atlas ring around the dens, the convex articular surfaces of C1, and their loose capsules, which allow sliding on C2 facets and prevent mutual contact. The first 30° of the axial rotation is performed by the inferior oblique muscle, which creates a backward translation of the atlas. The sternocleidomastoid and splenium capitis muscles, due to their opposite actions and oblique orientation, allow maximal rotation and stability. The tension generated on the alar ligaments controls the range of movement. Moreover, the transverse ligament limits the anterior displacement of the atlas. Failure of this ligament results in an increase atlantodental space (over 3 mm) or a reduction of the distance between the posterior surface of the odontoid and the posterior ring of C1 (below 13 mm) [2].

**Video 8:** Volumetric model showing the kinematics of the CCJ

## Conclusions

Understanding the three-dimensional relationship of the structures located in the CCJ and their kinematics is crucial for the proper identification and treatment of congenital and acquired craniospinal pathologies. Moreover, several lines and angles have been described to reveal the correct alignments of the different components of the CCJ and to establish the degrees of instability. The use of VMs for the depiction the CCJ’s multiple layers allows us to explore the complex anatomy of this region in 360-degrees, thereby facilitating pre-operative planning and reducing surgical difficulties.

## References

[1] Lopez AJ, Scheer JK, Leibl KE, Smith ZA, Dlouhy BJ, Dahdaleh NS (2015). Anatomy and biomechanics of the craniovertebral junction. Neurosurg Focus.

[2] Izzo R, Ambrosanio G, Cigliano A, Cascone D, Gallo G, Muto M (2007). Biomechanics of the spine III. The cranio-cervical junction. Neuroradiol J.

[3] Rubio RR, Shehata J, Kournoutas I (2019). Construction of neuroanatomical volumetric models using 3-dimensional scanning techniques: technical note and applications. World Neurosurg.

[4] Amankulor N, Gould G, Abbed KM (2011). Occipital-cervical and upper cervical spine fractures. The Comprehensive Treatment of the Aging Spine.

[5] Weiglein AH, Schmidberger HR (1998). The radio-anatomic importance of the colliculus atlantis. Surg Radiol Anat.

[6] Kim MS (2015). Anatomical variant of atlas: arcuate foramen, occpitalization of atlas, and defect of posterior arch of atlas. J Korean Neurosurg Soc.

[7] Cramer GD (2014). The cervical region. Clinical Anatomy of the Spine, Spinal Cord, and Ans (Third Edition).

[8] Tayebi Meybodi A, Gandhi S, Preul MC, Lawton MT (2018). The subatlantic triangle: gateway to early localization of the atlantoaxial vertebral artery. J Neurosurg Spine.

[9] La Rocca G, Altieri R, Ricciardi L, Olivi A, Della Pepa GM (2017). Anatomical study of occipital triangles: the 'inferior' suboccipital triangle, a useful vertebral artery landmark for safe postero-lateral skull base surgery. Acta Neurochir (Wien).

[10] Cohen MA, Evins AI, Lapadula G, Arko L, Stieg PE, Bernardo A (2017). The rectus capitis lateralis and the condylar triangle: important landmarks in posterior and lateral approaches to the jugular foramen. J Neurosurg.

[11] Hamada T, Usami A, Kishi A, Kon H, Takada S (2015). Anatomical study of phrenic nerve course in relation to neck dissection. Surg Radiol Anat.

[12] Cesmebasi A, Muhleman MA, Hulsberg P, Gielecki J, Matusz P, Tubbs RS, Loukas M (2015). Occipital neuralgia: anatomic considerations. Clin Anat.

[13] Schroeder GD, Hsu WK (2013). Vertebral artery injuries in cervical spine surgery. Surg Neurol Int.

[14] Cengiz SL, Cicekcibasi A, Kiresi D, Kocaogullar Y, Cicek O, Baysefer A, Buyukmumcu M (2009). Anatomic and radiologic analysis of the atlantal part of the vertebral artery. J Clin Neurosci.

[15] Smoker WR (1994). Craniovertebral junction: normal anatomy, craniometry, and congenital anomalies. Radiographics.

[16] Giammalva GR, Iacopino DG, Graziano F (2019). Surgical highways to the craniovertebral junction: Is it time for a reappraisal?. Acta Neurochir Suppl.

[17] Kwong Y, Rao N, Latief K (2011). Craniometric measurements in the assessment of craniovertebral settling: are they still relevant in the age of cross-sectional imaging?. AJR Am J Roentgenol.

[18] Tang C, Li GZ, Liao YH, Tang Q, Ma F, Wang Q, Zhong J (2019). Importance of the occipitoaxial angle and posterior occipitocervical angle in occipitocervical fusion. Orthop Surg.

[19] Guo Q, Ni B, Yang J, Liu K, Sun Z, Zhou F, Zhang J (2011). Relation between alignments of upper and subaxial cervical spine: a radiological study. Arch Orthop Trauma Surg.

[20] Visocchi M, Barbagallo G, Pascali VL (2017). Craniovertebral junction transanasal and transoral approaches: reconstruct the surgical pathways with soft or hard tissue endocopic lines? This is the question. Acta Neurochir Suppl.

